# Editorial: The Depth and Complexity of Unconscious Processing

**DOI:** 10.3389/fnhum.2021.791589

**Published:** 2021-11-22

**Authors:** Shen Tu, Jerwen Jou, Guang Zhao, Jun Jiang

**Affiliations:** ^1^Department of Psychology, School of Public Administration, Guizhou University of Finance and Economics, Guiyang, China; ^2^Department of Psychological Science, University of Texas – Rio Grande Valley, Edinburg, TX, United States; ^3^Faculty of Psychology, Tianjin Normal University, Tianjin, China; ^4^Department of Basic Psychology, School of Psychology, Third Military Medical University, Chongqing, China

**Keywords:** unconsciousness, unconscious perception, unconscious thought, unconscious priming, unconscious integration

The study of unconscious information processing mechanism is very important to the development of the science of human consciousness. By studying unconscious processing and comparing it with consciousness processing, we can better understand the ways unconsciousness works and the origin of conscious processing. The scope and limitations of unconscious processing have been discussed for more than a century, but no consensus has been reached for a long time. Some researchers believe that the scope of information that unconscious processing can reach is very limited, and only simple stimuli can be processed unconsciously, while others believe that any types of information can be processed unconsciously. Although the hypothesis that the scope of unconscious processing is equivalent to that of the conscious processing remains to be verified, with the deepening of the study of the unconsciousness, the idea that unconscious processing is limited to simple types of information is questioned. The papers included in this Research Topic “The Depth and Complexity of Unconscious Processing” provide some new evidence with respect to the complexity of unconscious processing. Here, we first present a brief overview of the contents of the present research topic.

The relationship between attention and awareness has been debated. Two papers in this special issue investigated this problem. Baier et al. demonstrated that stimulus-driven attention and awareness can operate independently from one another. Using a method of a combination of metacontrast-masking and stimulus-driven attention, they manipulated target visibility by varying the time interval between the target and the mask, and controlled stimulus-driven attention capture by presenting the masked target either as a color-singleton, or as a non-singleton together with a distractor singleton elsewhere. The results showed that the target visibility masking interval and the singleton manipulation affected the accuracy and subjective target awareness independently, supporting the concept of independent roles of stimulus-driven attention and awareness. Thus, although attention is often entangled in the relationship with consciousness, the dissociation of attention from consciousness makes unconscious attention possible. Güldener et al. manipulated the tilt-based attentional selection bias by presenting uneven proportions of the masked left-tilted and right-tilted gratings. By combining signal detection theory and subjective measures of awareness, they revealed that there was a weighted attentional bias toward selecting the relatively more frequent left- or right-tilted grating. More importantly, the response on the unaware weighted switch trials was significantly slower than that on the unaware repeat trials. This indicated that subliminal stimuli could trigger the reallocation of history-guided visual selection weights.

In the study of unconsciousness, although there is a dispute over the standard definition of the unconsciousness, the existence of unconscious processing is a reliable phenomenon. In this special topic issue, Rohr and Wentura reviewed non-conscious emotional information processing, and reached a similar conclusion. They also give dual avenues for better unconsciousness research to researchers: (a) improve the (methodological) criteria for what constitutes (non)-consciousness, and (b) empirically improve the understanding of non-conscious emotion processing. The dual avenues for improving research in unconscious information processing can also be applied to other areas of unconscious processing, such as unconscious symbolic, semantic, and social information processing.

Three other papers dealt with the complexity of unconscious processing, and the involvement of unconscious processing in other factors including, for example, unconscious integration between multiple unconscious stimuli. Jiménez-Ortega et al. manipulated, in a sentence, a masked number anomaly between a masked adjective and an unmasked noun, and a conscious number anomaly between an unmasked verb and an unmasked noun as well as the emotional valence of the masked adjective by using positive, negative, and neutral words. The results showed that syntactic components of brain waves LAN and P600 were observed for processing unconscious anomalies, which indicated the automatic nature of syntactic processing. Also, the unconscious emotional information modulated the conscious syntactic processing, indicating the flexible, adaptable, and context-dependent nature of the syntactic processing. Zhao et al. found that contexts followed by reward feedback could give rise to faster implicit learning. Hirschhorn et al. reviewed the relevant literature about unconscious integration (combining different signals into a coherent, unified whole) and put forth the “windows of integration” hypothesis. They concluded that the relevant studies provided compelling evidence for unconscious integration, albeit with scope limits in time, space, and semantic distance. The “windows of integration” hypothesis provided a distinction between conscious integration and unconscious awareness.

As a summary, first, the dual avenues proposed by Rohr and Wentura are a promising approach for addressing the shortcomings of the methods in the study of unconscious information processing. Secondly, for specific unconscious processing, even under relatively strict unconscious standards, there were studies that proved the existence of unconscious processing, especially regarding the recent evidence for relatively complex unconscious processing, such as unconscious integration.

If we take the existence of unconscious processing for granted, in what directions should future unconscious studies be going? We suggest that the unconscious contents to be researched may go beyond the scope of unconscious perception into unconscious thoughts. For example, in the studies of the incubation effect on creativity, a distracting, irrelevant task was usually inserted into an ongoing creative task to interrupt the latter for a short time period. Compared with the no-interruption group, the interrupted group achieved greater creativity in the creative task. The beneficial effect of interruption was attributed to unconscious processing of target task (Gilhooly et al., 2013). The unconscious process involved in creativity studies can be characterized as “not having explicit knowledge of” the goal state, which was also indicated in Zhao et al.'s study in this Research Topic, although other studies in this issue focused on “the lack of perceptual awareness.” The unconscious processes underlying the incubation period (Smith, 1995) prior to the arrival of the insight to a problems solution are not completely understood and worth further investigation. In an academic survey (Francken et al., 2021), the “conscious content” was defined as *what one is conscious of, when one is conscious* (an attentive, waking mental state). Maybe the unconscious content can be partly defined as the opposite of the conscious one, i.e., *what one is not conscious of, when one is conscious* that should include both implicit knowledge and a state of absence of perceptual awareness. In this context, future unconsciousness studies may need to focus on how multiple unconscious processes are involved to impact a behavior, as well as the interaction between the unconscious and conscious processes.

## Author Contributions

ST, JJo, and GZ wrote the Editorial. ST, JJo, and JJi revised and completed the Editorial. All authors contributed to the article and approved the submitted version.

## Conflict of Interest

The authors declare that the research was conducted in the absence of any commercial or financial relationships that could be construed as a potential conflict of interest.

## Publisher's Note

All claims expressed in this article are solely those of the authors and do not necessarily represent those of their affiliated organizations, or those of the publisher, the editors and the reviewers. Any product that may be evaluated in this article, or claim that may be made by its manufacturer, is not guaranteed or endorsed by the publisher.
